# Effects of 12 Weeks of Chromium, *Phyllanthus emblica* Fruit Extract, and Shilajit Supplementation on Markers of Cardiometabolic Health, Fitness, and Weight Loss in Men and Women with Risk Factors to Metabolic Syndrome Initiating an Exercise and Diet Intervention: A Randomized Double-Blind, Placebo-Controlled Trial †

**DOI:** 10.3390/nu17122042

**Published:** 2025-06-19

**Authors:** Victoria Martinez, Kay McAngus, Broderick L. Dickerson, Megan Leonard, Elena Chavez, Jisun Chun, Megan Lewis, Dante Xing, Drew E. Gonzalez, Choongsung Yoo, Joungbo Ko, Heather Rhodes, Hudson Lee, Ryan J. Sowinski, Christopher J. Rasmussen, Richard B. Kreider

**Affiliations:** Exercise & Sport Nutrition Laboratory, Human Clinical Research Facility, Department of Kinesiology and Sports Management, Texas A&M University, College Station, TX 77843, USA; victoria.jenkins@tamu.edu (V.M.); kirsten.nottingham@cpcmed.org (K.M.); dickersobl5@email.tamu.edu (B.L.D.); meganleonard10@tamu.edu (M.L.); ebchavez_10@tamu.edu (E.C.); chunjs3112@tamu.edu (J.C.); dantexing@tamu.edu (D.X.); dg18@tamu.edu (D.E.G.); choongsungyoo@tamu.edu (C.Y.); joungboko10@tamu.edu (J.K.); hr3740@pcom.edu (H.R.); hudson.qml@tamu.edu (H.L.); rjs370@tamu.edu (R.J.S.); crasmussen@tamu.edu (C.J.R.)

**Keywords:** obesity, diet, insulin sensitivity, inflammation, blood lipids, metabolic syndrome, functional capacity, quality of life

## Abstract

**Background:** Exercise and nutritional interventions are often recommended to help manage risk related to metabolic syndrome (MetSyn). The co-ingestion of *Phyllanthus emblica* (PE) with trivalent chromium (Cr) has been purported to improve the bioavailability of chromium and enhance endothelial function, reduce platelet aggregation, and help manage blood glucose as well as lipid levels. Shilajit (SJ) has been reported to have anti-inflammatory, adaptogenic, immunomodulatory, and lipid-lowering properties. This study evaluated whether dietary supplementation with Cr, PE, and SJ, or PE alone, during an exercise and diet intervention may help individuals with risk factors to MetSyn experience greater benefits. **Methods**: In total, 166 sedentary men and women with at least two markers of metabolic syndrome participated in a randomized, placebo-controlled, parallel-arm, and repeated-measure intervention study, of which 109 completed the study (48.6 ± 10 yrs., 34.2 ± 6 kg/m^2^, 41.3 ± 7% fat). All volunteers participated in a 12-week exercise program (supervised resistance and endurance exercise 3 days/week with walking 10,000 steps/day on non-training days) and were instructed to reduce energy intake by −5 kcals/kg/d. Participants were matched by age, sex, BMI, and body mass for the double-blind and randomized supplementation of a placebo (PLA), 500 mg of PE (PE-500), 1000 mg/d of PE (PE-1000), 400 µg of trivalent chromium (Cr) with 6 mg of PE and 6 mg of SJ (Cr-400), or 800 µg of trivalent chromium with 12 mg of PE and 12 mg of SJ (Cr-800) once a day for 12 weeks. Data were obtained at 0, 6, and 12 weeks of supplementation, and analyzed using general linear model multivariate and univariate analyses with repeated measures, pairwise comparisons, and mean changes from the baseline with 95% confidence intervals (CIs). **Results**: Compared to PLA responses, there was some evidence (*p* < 0.05 or approaching significance, *p* > 0.05 to *p* < 0.10) that PE and/or Cr with PE and SJ supplementation improved pulse wave velocity, flow-mediated dilation, platelet aggregation, insulin sensitivity, and blood lipid profiles while promoting more optimal changes in body composition, strength, and aerobic capacity. Differences among groups were more consistently seen at 6 weeks rather than 12 weeks. While some benefits were seen at both dosages, greater benefits were more consistently observed with PE-1000 and Cr-800 ingestion. **Conclusions**: The results suggest that PE and Cr with PE and SJ supplementation may enhance some exercise- and diet-induced changes in markers of health in overweight individuals with at least two risk factors to MetSyn. Registered clinical trial #NCT06641596.

## 1. Introduction

As adults age, they often become less active, gain fat mass, and have difficulty managing blood glucose and lipid levels, thereby increasing their risk of type 2 diabetes mellitus and cardiometabolic disease. Metabolic syndrome (MetSyn) is defined as the presence of at least three risk factors, including abdominal obesity, hypertension, glucose intolerance, high triglycerides, and low high-density lipoprotein levels [1]. Exercise and nutritional interventions that promote fat loss, help manage blood glucose and lipids, and reduce oxidative stress and inflammation are often recommended to reduce the risk of cardiometabolic disease and MetSyn before considering pharmacological management [1,2,3,4]. Understanding how exercise, diet, and nutritional interventions may affect cardiometabolic risk factors can help individuals and clinicians make informed decisions about their impact on health and cardiometabolic risk factors. This study evaluated whether dietary supplementation of three nutrients purported to affect cardiometabolic risk factors would enhance outcomes to an exercise, diet, and weight loss program intervention in sedentary individuals with MetSyn risk factors.

*Phyllanthus emblica* (also known as emblic, balakka, myrobalan, and Indian gooseberry) is a tree native to Southern Asia that contains phenolic compounds, flavonoids, alkaloids, terpenoids, phytosterols, and other compounds [5] that have antioxidant [6,7,8,9], anti-inflammatory [5,10], and cardioprotective properties [5]. *Phyllanthus emblica* has also been reported to increase nitrous oxide levels [7,11,12], enhance endothelial function [8], decrease platelet aggregation [13], and improve blood glucose [10,13,14] and lipids [10,13]. Shilajit (also known as mumijo, mumie, or mamlayi) is a naturally occurring mineral substance found in rock crevices in mountainous regions, particularly in Central Asia [15]. Shilajit has antioxidant [15,16,17,18], anti-inflammatory [17,18,19] and adaptogenic properties [20,21,22] and has been used in traditional Indian medicine to manage hypertension [15], inflammation [17,19,23], hyperlipidemia [15,18], and diabetes [15,24]. Chromium has been purported to improve insulin sensitivity and help manage blood glucose levels, particularly in individuals with glucose intolerance and/or type 2 diabetes [25,26,27,28,29,30,31,32,33,34,35]. There is also some evidence that chromium supplementation may promote fat loss and promote more favorable changes in body composition during exercise training [36,37,38,39,40,41,42]. However, the bioavailability, preservation of the bioactive chromium III complexes, and minimization of the conversion of chromium III to VI may influence the efficacy of chromium supplements [4,43]. Theoretically, co-ingesting chromium, PE, and/or SJ during an exercise and weight loss program could promote greater glucose control, blood lipids, and/or body composition benefits, thereby helping manage cardiometabolic and MetSyn risk factors. However, more research is needed to evaluate the independent and potentially synergistic effects of Cr, PE, and SJ supplementation in addition to exercise and diet interventions before conclusions can be drawn.

The study investigated the efficacy and safety of the dietary supplementation of a trivalent chromium (Cr) with *Phyllanthus emblica* (PE) and Shilajit (SJ) in sedentary and overweight men and women with at least two risk factors to MetSyn, initiating an exercise and weight loss program. The rationale in comparing these nutrients was to determine whether co-ingesting chromium, PE, and SJ provides greater effects on MetSyn risk factors than PE alone, and whether there is a dose effect. We hypothesized that supplementing the diet with chromium, PE, and SJ would promote greater improvements in markers of health and training adaptations in this population than exercise and training alone. Additionally, there may be additive benefits from the inclusion of trivalent chromium with PE and SJ.

## 2. Methods

### 2.1. Research Design

**Figure 1:**
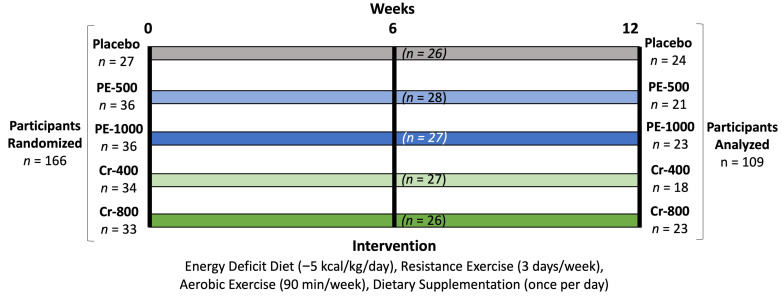
Overview of experiment study design. PE = *Phyllanthus emblica*, Cr = chromium.

Figure 1 shows the experimental design used in this study. This study was approved by the Human Protection Institutional Review Board (IRB2019-1714F, approved 16 May 2019) following the Declaration of Helsinki ethical standards for conducting human participant research. This clinical trial was also registered by clinicaltrials.gov (#NCT06641596, posted 15 October 2024). This paper is an extended version of abstracts presented at the 2024 International Society of Sports Nutrition conference [44]. All participants signed written consent forms and participated in a 12-week supervised exercise program and energy-reduced diet program. The independent variable was nutritional supplementation. Primary endpoints included markers of endothelial function (i.e., flow-mediated dilation, venous occlusion, and platelet aggregation) and cardiometabolic risk factors (i.e., fasting glucose, HbA1c, inflammatory markers, and lipid profiles). Secondary endpoints included changes in diet, weight, body composition, resting energy expenditure, resting hemodynamics, maximal aerobic capacity, changes in musculoskeletal strength and endurance, markers of catabolism/anabolism, mood states, quality of life assessments, and assessment of health and side effects.

### 2.2. Study Participants

Participant inclusion criteria were as follows: (1) Sedentary men and women aged 30–65 with a body mass index (BMI) > 30 and/or percent body fat > 30%. (2) Evidence of at least one additional risk factor to MetSyn (i.e., fasting triglycerides > 150 mg/dL or treatment for high triglycerides, high-density lipoprotein (HDL) < 40 mg/dL in males and < 50 mg/dL in females, resting systolic blood pressure (SBP) > 130 mmHg or diastolic blood pressure (DBP) > 85 mmHg, or treatment of previously diagnosed hypertension, blood glucose ≥ 100 mg/dL, or previous diagnosis for type I or II diabetes). (3) Medical clearance to participate in moderate to intense exercise training and testing. (4) Absence of limiting musculoskeletal injury that would prevent participation in a general fitness program. (5) Given voluntary, written, and informed consent to participate in the study. Participants were not allowed to participate in the study if they (1) were taking or had taken nitrous oxide or anti-inflammatory type supplements or medications within one month of the start of the study; (2) had uncontrolled hypertension, triglycerides > 500 mg/dL, elevated liver enzymes three times the upper limit, and serum creatinine > 1.5 mg/dL); (3) did not receive medical clearance from their physician to participate in the study and exercise program; or (4) were pregnant or planned to become pregnant. Participants had to complete at least 90% of the training sessions.

**Figure 2:**
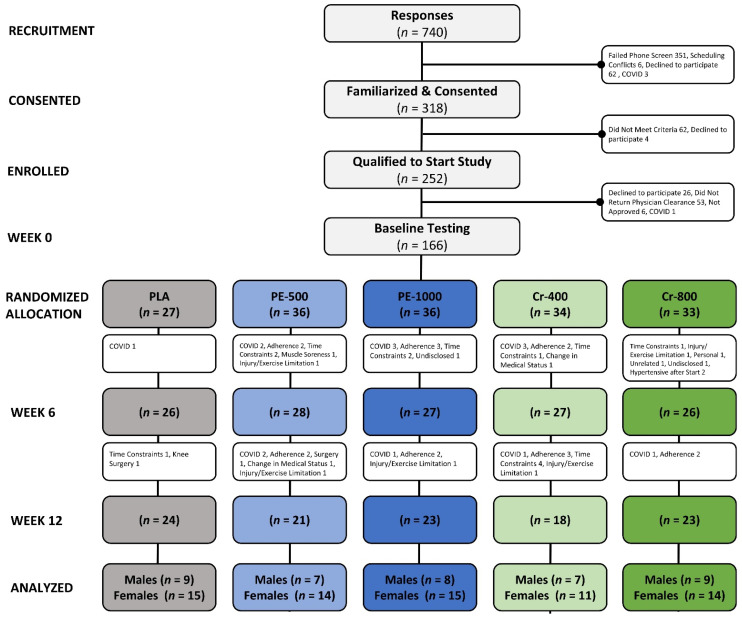
Consolidated Standards of Reporting Trials (CONSORT) flow chart. PLA = placebo, PE = Phyllanthus emblica, Cr = chromium, and *n* = sample size.

A Consolidated Standards of Reporting Trials (CONSORT) diagram is shown in Figure 2. Data collection began in the summer of 2019 and continued through the fall of 2023. The COVID-19 epidemic and resulting infection control procedures affected participant recruitment and compliance. A total of 740 prospective participants responded to research advertisements and were evaluated for eligibility: 318 were familiarized and consented to participate, 252 qualified and agreed to participate, and 166 participants obtained physician clearance and began the study. Participants were matched according to age, sex, and BMI into one of four treatment groups that included a placebo (PLA), 500 mg of PE (PE-500), 1000 mg of PE (PE-1000), 400 µg of Cr with 6 mg of PE and 6 mg of SJ (Cr-400), or 800 µg of Cr with 12 mg of PE and 12 mg of SJ (Cr-800). A total of 24 (15 females, 9 males), 23 (15 females, 8 males), 21 (14 females, 7 males), 18 (11 females, 7 males), and 23 (14 females, 9 males) participants completed the treatments, respectively. Reasons for study discontinuation included COVID-19 exposure (14), non-compliance/adherence (*n* = 16), scheduling/time constraints (*n* = 16), unrelated injury (*n* = 4), change in medical status after unrelated annual physicals (*n* = 2), unrelated surgery (*n* = 2), muscle soreness (*n* = 1), car accident (*n* = 1), and undisclosed (*n* = 2). One female and one male in the Cr-800 treatment arm experienced hypertension in response to the initiation of the exercise training protocol and were withdrawn.

### 2.3. Familiarization

Respondents to research advertisements were contacted to determine their eligibility for a familiarization session. Individuals meeting the initial phone screening eligibility were invited to the lab, where an overview of the study was provided. Those interested in participating in the study signed informed consent statements, completed health questionnaires, donated a fasting blood sample, and had their height, weight, resting heart rate, and resting blood pressure determined. This information was used to assess their eligibility to participate in the study. Volunteers then had the diet and exercise training program explained to them. Those meeting the eligibility requirements were asked to obtain approval from their physician to participate in the study. Once physician clearance was procured, participants were scheduled for baseline testing.

### 2.4. Experimental Session Testing Protocol

**Figure 3:**
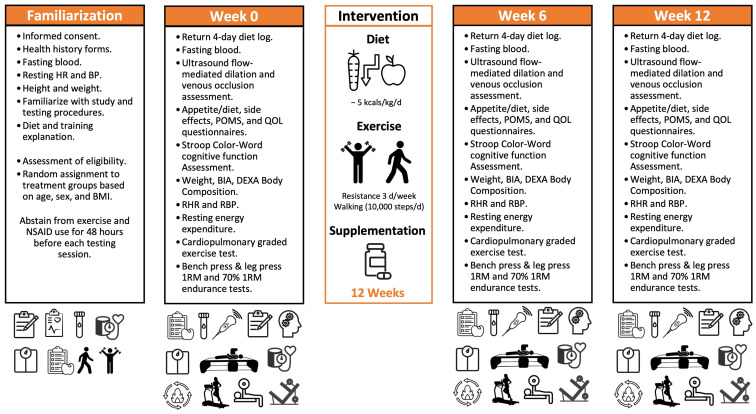
Study timeline and testing sequence. HR = heart rate, BP = blood pressure, BMI = body mass index, NSAID = non-steroidal anti-inflammatory drug, POMS = Profile of Mood States, QOL = quality of life, 1RM = one-repetition maximum, BIA = bioelectrical impedance, and DEXA = dual-energy X-ray absorptiometer.

Figure 3 presents the research timeline for testing. Participants completed a familiarization and three testing sessions. Those found eligible to participate in the study during the familiarization session signed informed consent statements and were scheduled for the initial experimental session. Participants recorded their dietary intake for four days, refrained from intense exercise for 48 h, and fasted for 12 h before reporting to testing sessions. For baseline testing, participants signed informed consent statements, completed health history questionnaires, and donated a fasting blood sample. They then had their height, weight, resting heart rate, and resting blood pressure determined. During the baseline testing, participants returned a 4-day food log, had ultrasound flow-mediated dilation and venous occlusion assessments performed, completed appetite/diet, side effects, Profile of Mood States (POMS), and quality of life (QOL) questionnaires, and performed a Stroop Color–Word cognitive function assessment. They then had their weight, total body water, dual-energy X-ray absorptiometer (DEXA) body composition, resting heart rate and blood pressure, and resting energy expenditure determined. Volunteers then performed an incremental, symptom-limited, maximal cardiopulmonary exercise test (GPXT), one-repetition maximum (1RM) bench press and leg press tests, and bench press and leg press muscular endurance at 70% of their 1RM. Participants were instructed to reduce energy intake by about five kcals/day while participating in the exercise training and dietary supplement program.

### 2.5. Randomization

All volunteers participated in the same energy-deficit diet and exercise program described below. Participants were matched within 5 kg cohorts as closely as possible based on age, BMI, and sex, and randomly assigned to ingest the PLA, PE-500, PE-1000, Cr-400, or Cr-800 treatments once daily for 12 weeks.

### 2.6. Exercise Training

Participants followed a supervised exercise program (3 days/week for 12 weeks) that included resistance and cardiovascular exercise. Training workloads were monitored and recorded on exercise cards. During COVID-19, participants had their forehead temperature determined and completed a survey assessing symptoms and known exposure before entering the facility. Participants exercised wearing surgical masks and face shields and/or with partitions placed between exercise modalities to maintain adequate distance between participants. Additionally, equipment was cleaned between uses to minimize the potential for virus transmission. The resistance training program consisted of 11 exercises emphasizing all major muscle groups. Participants performed three sets of 10 repetitions with about 2 min of rest between each set and exercise. Participants were encouraged to increase the weight lifted once they completed the prescribed number of repetitions for each set. Cardiovascular training involved treadmill walking, stationary cycling, or outside running/walking for 20 min at 60–80% heart rate reserve (HRR). Workloads were progressed to maintain heart rate within this range. To maintain compliance, participants needed to complete 90% (32/36 sessions) of exercise sessions [45]. Participants were encouraged to walk 10,000 steps/day, 3 days per week on non-training days. Step count was monitored via phones or clip-on pedometers (BATAUU, Shenzhen, China).

### 2.7. Diet Modification

Participants were given a list of high-energy-containing foods and asked to reduce energy intake by about five kcal/kg/day. Meal plan examples and a list of food and beverage exchanges were provided as previously described [46]. It was explained to participants how to reduce energy consumption and record dietary intake by a research assistant, with compliance assessed weekly during training sessions and at each testing session.

### 2.8. Dietary Supplementation

Volunteers were categorized in 5 kg weight intervals and matched by age, sex, and BMI to other individuals within that weight range for random assignment to treatment groups. Supplements were prepared in white 00-sized capsules (Capsugel, Lonza, Cambridge, MA, USA) and coded bottles for double-blind administration. The PLA consisted of two capsules containing 400 mg of microcrystalline cellulose (Vivapur^®^ 102, JRS Pharma LP, Patterson, NJ, USA, Batch #V102C3K33), 24 mg of croscarmellose sodium (Vivasol^®^, JRS Pharma LP, Patterson, NJ, USA, Batch #7111307247), 6 mg of silicon dioxide (Mutchler Inc., Harrington Park, NJ, USA, Batch #304091600), and 6 mg of magnesium stearate Kosher (Mutchler Inc., Harrington Park, NJ, USA, Batch #C416266), and packaged by Natreon, Inc., New Brunswick, NJ, USA. The PE-500 supplements consisted of 500 mg of PE (Sakti Naturals, Villupuram, India, Batch #CA201703010), 110 mg of microcrystalline cellulose (Vivapur^®^ 102, JRS Pharma LP, Patterson, NJ, USA, Batch #V102C3K33), croscarmellose sodium (Vivasol^®^, JRS Pharma LP, Patterson, NJ, USA, Batch #7111307247), 6 mg of silicon dioxide (Mutchler Inc., Harrington Park, NJ, USA, Batch #304091600), and 6 mg of magnesium stearate Kosher (Mutchler Inc., Harrington Park, NJ, USA, Batch #C416266), with one capsule of PLA. The PE-1000 supplements consisted of two capsules of PE-500. The Cr-400 supplement contained 20 mg of Crominex^®^ (Natreon Inc., New Brunswick, NJ, USA), 219 mg of microcrystalline cellulose (Vivapur^®^ 102, JRS Pharma LP, Patterson, NJ, USA, Batch #V102C3K33), 10 mg of croscarmellose sodium (Vivasol^®^, JRS Pharma LP, Patterson, NJ, USA, Batch #7111307247), and 1.25 mg of magnesium stearate Kosher (Mutchler Inc., Harrington Park, NJ, USA, Batch #C416266), with one PLA capsule. The Cr-800 group consumed two capsules of the Cr-400 supplement. Each company providing source materials provided a certificate of analysis, and the supplement contents were packaged and verified for content by Natreon Inc., New Brunswick, NJ, USA. Participants were instructed to ingest the supplements once daily after breakfast (about 8 AM). Supplement compliance was encouraged via email and phone communication and verified at each testing session.

## 3. Procedures

### 3.1. Diet Assessment

Participants recorded their dietary intake on food logs or by using the MyFitnessPal (MyFitnessPal, Inc., Baltimore, MD, USA) phone application [47]. Diet was analyzed using Food Processor Nutrition Analysis version 11.14.9 of software (ESHA Nutrition Research, Salem, OR, USA) [48,49].

### 3.2. Training Volume Assessment

Participants recorded the amount of weight lifted and repetitions performed for each set of exercises on workout cards during supervised training sessions. The training volume for each exercise was calculated by multiplying the weight lifted by the number of repetitions performed for each set and exercise. The upper body training volume was determined by adding all upper body exercises to determine a total volume from weeks 0–6 and 6–12. Similarly, lower body lifting volume represented the sum of all lower extremity exercises. Total lifting volume was calculated as the sum of all lifts during each training period. Participants recorded their heart rate and the distance completed on the treadmill during supervised workouts. Participants recorded step counts on non-training days. This information was used to determine the average step count on non-training days.

### 3.3. Resting Energy Expenditure Assessment

A calibrated ParvoMedics TrueOne 2400 Metabolic Measurement System (ParvoMedics Inc., Sandy, UT, USA) was used to assess resting energy expenditure by using the procedures previously described [46,50,51,52,53,54,55].

### 3.4. Body Composition, Total Body Water, and Resting Hemodynamics

Body mass and height were determined by using a Health-O-Meter Professional 500KL scale (Pelstar LLC, Alsip, IL, USA). Total body water was estimated by using an Impedimed SFB7 multi-frequency bioelectrical impedance analyzer (Impedimed, Inc., Carlsbad, CA, USA). Body composition, excluding the cranium, was measured with a calibrated Hologic Discovery W DEXA (Hologic Inc., Waltham, MA, USA) using APEX Software, version 5.6.1.4 (APEX Corporation Software, Pittsburgh, PA, USA) [56,57]. This measurement has a C_V_ of 0.3–0.5% and a mean intraclass correlation of 0.98 in our lab [58]. After the DEXA scan, the resting heart rate and blood pressure were measured in the supine position using a digital heart rate monitor and blood pressure cuff (Connex^®^ ProBP^TM^ 3400; Welch Allyn, Tilburg, The Netherlands).

### 3.5. Pulse Wave Velocity Assessment

Pulse wave velocity (PWV) was assessed using the carotid–femoral method by using a standard ultrasonographic procedure with a GE Logiq-P6 ultrasound with Application Software R1.0.4 (GE Healthcare, Chicago, IL, USA) [59]. Participants were asked to lay supine on a table. Electrodes were attached at the right arm, left arm, and right leg locations to obtain R-wave readings by using an ultrasound ECG. Participants were asked to turn their heads to the left to allow access to the carotid artery. The 11L probe, placed on the neck parallel to the sternocleidomastoid muscle, was used to obtain an image of the carotid blood flow. Once a steady image of the arterial blood flow was obtained, a 6-beat heart rate was recorded by using the store function on the Logiq-P6 ultrasound. The 11L probe was placed on the participant’s hip/groin area to obtain the femoral recording. Upon locating the femoral arterial blood flow, a 6-beat heart rate was recorded. The distance between the location of the carotid arterial reading and the femoral arterial reading was recorded. Standard carotid–femoral calculations were used to calculate the final pulse wave velocity.

### 3.6. Flow-Mediated Dilation Assessment

Flow-mediated dilation (FMD) was assessed on the right arm by using a standard GE Logiq-P6 ultrasound with Application Software R1.0.4 (GE Healthcare, Chicago, IL, USA) using standard methods [60,61]. Participants were asked to lie supine on a table with their right arm extended to the side for 10 min before beginning the baseline measure. Using the 11L probe, the brachial artery was located using the bicep and medial epicondyle as reference points. Upon stabilizing the image of the brachial artery, a 60 s video was recorded for a baseline measure. Following the 60 s video, pressure was applied to the forearm using a manual blood pressure cuff at 200 mmHg to occlude blood flow for 5 min. Before releasing the blood pressure cuff, a stable view of the now-occluded brachial artery was obtained. At the 5 min mark, pressure was released from the cuff, and a 90 s video was recorded as a “deflation” measurement. The average diameter of the blood vessel at the baseline and after deflation was used to determine a percent change in diameter as an indicator of the health and function of the epithelial lining of the blood vessels. The reported percent change in diameter will be allometrically scaled to remove bias from the calculation [62].

### 3.7. Blood Collection and Analysis

Participants donated fasting venous blood samples at each testing session. Fasting blood was collected using standard procedures [63] by certified phlebotomists into BD Vacutainer^®^ serum separation tubes (SST, Becton, Dickinson and Company, Franklin Lakes, NJ, USA). Samples sat at room temperature (15 min) and were then centrifuged at 3500 rpm for 10 min using a Thermo Scientific Heraeus MegaFuge 40R Centrifuge (Thermo Electron North America LLC, West Palm Beach, FL, USA). Serum was aliquoted into microcentrifuge tubes and stored at −80 °C for subsequent analysis. Whole blood was collected into K2 ethylenediaminetetraacetic acid (EDTA) tubes (BD Vacutainer^®^, Becton, Dickinson and Company, Franklin Lakes, NJ, USA). The SST and EDTA samples were sent to the Clinical Pathology Laboratory (Austin, TX) to assess cell blood counts, serum blood profiles, hemoglobin A1C (HbA1c), and high-sensitive C-reactive protein (hsCRP). Serum insulin was determined using enzyme-linked immunoassay (ELISA) assay kits (Alpco Diagnostics, Salem, NH, USA) with a BioTek Epoch 2 plate reader with BioTek Gen 5 software (BioTek Instruments, Winooski, VT, USA). The glucose/insulin ratio (GIR), homeostatic model assessment for insulin resistance (HOMA-IR), and quantitative insulin sensitivity check index (QUICKI) were used to assess insulin sensitivity [64]. Cytokines were determined from stored samples by using a Cytokine Human Magnetic 10-plex Panel kit with a Luminex™ 200™ Instrument System (ThermoFisher Scientific, Vienna, Austria) by using xPONENT^TM^ version 3.1 software. Inter-assay and intra-assay coefficients of variation (CVs) ranged between 2% and 18% and 3% and 10%, respectively. Outliers were identified by using the Grubbs procedure [65]. Platelet aggregation was determined from whole-blood samples by using a CHRONO-LOG^®^ Model 700 Whole Blood/Optical Lumi-Aggregometer with AGGRO/LINK^®^8 and vW Cofactor Software, version 8 (Havertown, PA, USA) according to manufacturer specifications.

### 3.8. Exercise Assessment

Participants performed a submaximal exercise test to 80% of HRR using the Bruce protocol on a treadmill (TrackMaster 425, Newton, KS, USA) [66]. The time to achieve 80% of HRR was recorded. Expired ventilation, oxygen, and carbon dioxide content were measured using previously described procedures [3,46,50,51,67,68,69] by using a TrueOne 2400 metabolic measurement device (ParvoMedics Inc., Sandy, UT, USA) and a Cardio-Card version 7.2 electrocardiograph (NasifF Associates, Brewerton, NY, USA). Bench press and leg press 1RM and 70% of 1RM tests were performed using standard procedures [70] on a bench and hip sled/leg press (Nebula Fitness, Versailles, OH, USA).

### 3.9. Quality of Life

The Short Form Health Survey version 2 (SF-36v2) was used to assess perceptions about QOL and functional capacity [71]. The test–retest reliability of this survey has been reported to have strong correlations on all domains (r = 0.81–0.95) [72,73].

### 3.10. Diet Satisfaction

Subjective ratings of diet satisfaction were determined using a Likert scale that asked participants to rate appetite, hunger, food satisfaction, feelings of fullness, energy levels, and diet quality. The scale ranged from 0 (none) to 10 (most). Participants were asked to make a mark along a continuous rating scale (divided at 0.1 intervals) to record ratings.

### 3.11. Side Effects

Side effects were evaluated by using a survey on the frequency and severity of subjective side effects, as previously described [50,74,75]. The C_V_ of questions ranged from 1% to 3%, with intraclass correlations between 0.6 and 0.88 [50,74,75].

### 3.12. Statistical Analysis

A comprehensive as well as statistical analysis [76,77,78] and clinical assessment of mean changes from the baseline [76,77,78,79,80,81,82] were performed by using previously described methods [83]. Sample size was determined based on our prior research that evaluated the effects of exercise, diet, and nutritional interventions on weight loss and markers of MetSyn [67,68,69,84,85,86,87], assuming an 80% power with a 5–10% standard deviation to the mean and a 5–10% improvement in primary outcomes. This analysis revealed that a sample size of 20–25 per group in a parallel-arm study was sufficiently powered to detect significant differences between treatment groups. The International Business Machines (IBM) Statistical Package for the Social Sciences (SPSS) version 29 statistical analysis software (IBM Corp., Armonk, NY, USA) was used to analyze data. Numerical missing data, if there were any, were replaced using the series’ means [88]. Missing ordinal survey responses, if there were any, were replaced using the most frequent response or value method [89]. Numerical data were analyzed by using a mixed-model general linear model (GLM) analysis of variance (ANOVA) with repeated measures. Mauchly’s test was used to assess sphericity, and the kurtosis statistic was used to assess normality. The Wilks’ Lambda and Greenhouse–Geisser univariate correction tests were used to adjust for F-value inflation [90,91]. Fisher’s Least Significant Difference (LSD) tests were used to assess pairwise comparisons at pre-planned contrasts of interest and as post hoc tests. The probability of a type I statistical error was 0.05 or less. Statistical trends were identified when *p*-values ranged between 0.05 and 0.10. Effect size was analyzed using partial eta squared (η_p_^2^), where values of 0.01 represented a small effect, 0.06 represented a medium effect, and 0.14 represented a large effect size [92]. Clinical significance was examined by assessing changes from the baseline with 95% confidence intervals (CIs) [80]. Chi-squared analysis was used to analyze categorical responses to surveys. Data are means and standard deviations (mean ± SD) or mean changes from the baseline (mean or percentage mean [LL, UL]).

## 4. Results

### 4.1. Participant Demographics

Table S1 presents participant demographics by group and sex. Participants were 48.6 ± 10 years, 169.5 ± 9 cm, 99.4 ± 20 kg, 34.6 ± 6 kg/m^2^, and 41.3 ± 7% fat. Multivariate GLM analysis revealed sex (*p* < 0.001, η_p_^2^ = 0.598), but no group × sex (*p* = 0.545, η_p_^2^ = 0.046), effects. Sex effects were seen for age (*p* = 0.013), height (*p* < 0.001), weight (*p* = 0.012), and percent body fat (*p* < 0.001). No group × sex effects were seen for the demographic data, although BMI (*p* = 0.062) tended to interact.

### 4.2. Energy and Macronutrient Intake

Table S2 presents absolute as well as relative energy and macronutrient intake analyzed for 94 participants that submitted analyzable food logs. Energy intake averaged 1669 kcals/d [1589, 1749] (17.4 kcals/kg/d [0.79, 0.88), which is consistent with diets shown to facilitate weight loss during exercise training in our prior weight loss intervention studies [3,45,46,51,69,75,86]. Some group and pairwise differences were observed among groups in terms of energy and macronutrient intake, primarily from the placebo and supplemented groups. However, no significant multivariate or univariate time or interaction effects were observed in terms of absolute or relative energy, carbohydrate, fat, or protein intake.

### 4.3. Physical Activity

Table S3 shows resistance training lifting volume and progression from the first to last six weeks of the study. GLM analysis revealed significant time effects (*p* < 0.001, η_p_^2^ = 0.888), while no interaction effects were observed (*p* = 0.462, η_p_^2^ = 0.037). Total lifting volume increased by 95% (*p* < 0.001) from the first to the last six weeks of training. Likewise, univariate analysis showed significant time effects in upper body, lower body, and total lifting volume (*p* < 0.001), with no group × time effects observed. Participants walked for 30 min at 60–80% of their heart rate reserve during supervised training days and recorded step counts on non-training days. Non-training step counts averaged 9241 ± 2232 steps per day, with no significant difference observed among groups (see Table S4).

### 4.4. Resting Energy Expenditure and Metabolism

**Figure 4:**
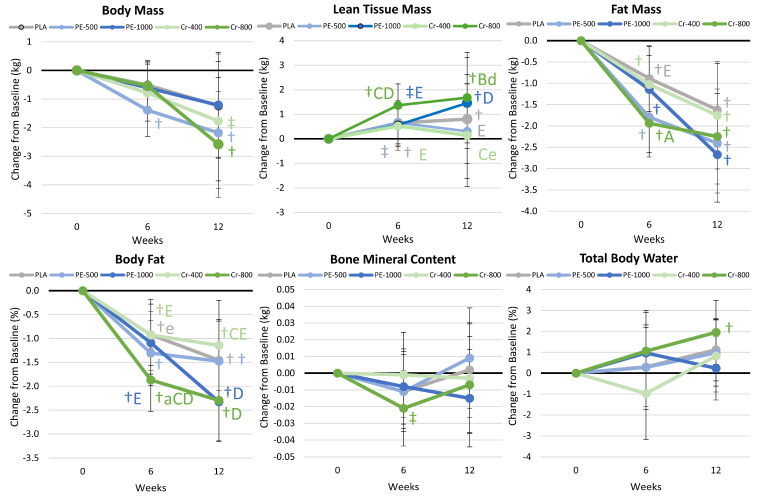
Body composition and body water results. Data are means and 95% confidence intervals. † is a *p* < 0.05 difference from the baseline (‡ = *p* > 0.05 to *p* < 0.10 difference). PLA = A, PE-500 = B, PE-1000 = C, Cr-400 = D, and Cr-800 = E. Lowercase letters = *p* < 0.05 difference. Uppercase letters = *p* > 0.05 to *p* < 0.10 difference.

Table S5 shows resting energy expenditure data. Overall, multivariate analysis revealed a significant time (*p* = 0.006, η_p_^2^ = 0.043), while no interaction effects were observed (*p* = 0.649, η_p_^2^ = 0.033). Univariate analysis indicated that the respiratory exchange ratio (*p* = 0.001, η_p_^2^ = 0.069) and carbohydrate oxidation (*p* = 0.001, η_p_^2^ = 0.070) decreased while fat oxidation (*p* = 0.001, η_p_^2^ = 0.070) increased over time, with no significant interaction effects. Figure 4 shows the mean change results. After 6 weeks of training, resting energy expenditure increased in the Cr-400 group. Training also promoted a reduction in resting carbohydrate oxidation and an increase in fat oxidation, with no significant differences observed among groups. Mean changes from the baseline are shown in Figure S1.

### 4.5. Cardiovascular Health and Fitness Results

Table S7 shows results for resting hemodynamic and peak aerobic capacity. Multivariate analysis revealed a significant time effect (*p* < 0.001, η_p_^2^ = 0.237), while no interaction effects were observed (*p* = 0.172, η_p_^2^ = 0.045). Univariate analysis indicated significant time effects in terms of resting heart rate (*p* = 0.002, η_p_^2^ = 0.057), resting diastolic blood pressure (*p* = 0.001, η_p_^2^ = 0.074), absolute peak oxygen uptake (*p* = 0.001, η_p_^2^ = 0.233), relative peak oxygen uptake (*p* = 0.001, η_p_^2^ = 0.246), and time to fatigue (*p* = 0.001, η_p_^2^ = 0.320) with no significant group × time effects observed. Figure S2 shows the mean changes from the baseline values. Resting heart rate and diastolic blood pressure decreased in most groups, with some differences observed among groups, although the differences were small. All groups increased their aerobic capacity and time to fatigue, with those in the Cr-400 group increasing to a greater degree than some other groups. However, none of these differences were significantly different than PLA responses. These findings indicate that the exercise intervention promoted significant improvement in aerobic capacity, but that supplementation did not provide additive benefits.

### 4.6. Muscular Strength and Endurance Results

Table S8 shows results for muscular strength and endurance. Multivariate analysis revealed a significant time effect (*p* < 0.001, η_p_^2^ = 0.467), while no interaction effects were observed (*p* = 0.214, η_p_^2^ = 0.045). Univariate analysis indicated significant time effects in resting bench press 1RM (*p* = 0.001, η_p_^2^ = 0.562), bench press endurance at 70% of 1RM (*p* = 0.001, η_p_^2^ = 0.413), leg press 1RM (*p* = 0.001, η_p_^2^ = 0.627), and leg press endurance (*p* = 0.001, η_p_^2^ = 0.297). A significant group × time effect was observed in leg press 1RM (*p* = 0.012, η_p_^2^ = 0.094). All groups gained strength and endurance, with bench press and endurance tending to be higher in the Cr-800 group. Figure S3 shows changes in upper and lower muscular strength and endurance. For all groups, 1RM strength and endurance increased. However, those in the PE-1000 group experienced significantly greater gains in 1RM strength than those in other groups. Although more research is needed, these findings suggest that PE-1000 supplementation may promote greater gains in muscular strength than PLA, PE-500, Cr-400, and Cr-800 supplementation, which should be further explored.

### 4.7. Body Composition Results

**Figure 5:**
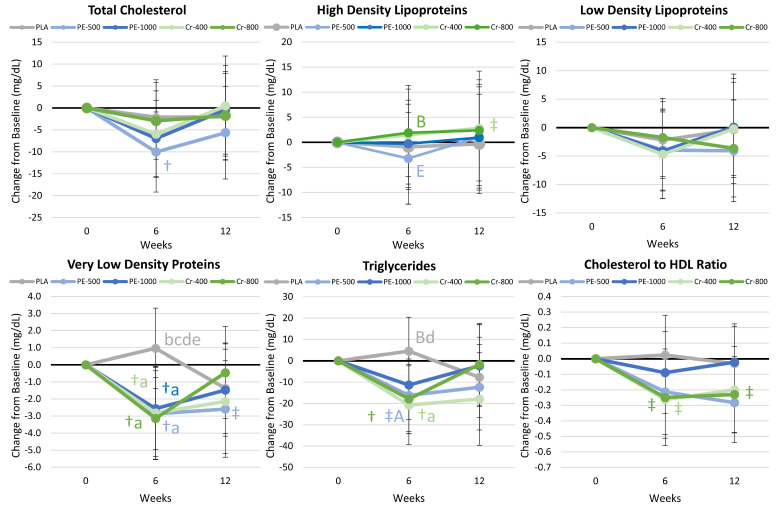
Changes in blood lipids. Data are means and 95% confidence intervals. HDL is high-density lipoprotein. † is a *p* < 0.05 difference from the baseline (‡ = *p* > 0.05 to *p* < 0.10 difference). PLA = A, PE-500 = B, PE-1000 = C, Cr-400 = D, and Cr-800 = E. Lowercase letters = *p* < 0.05 difference. Uppercase letters = *p* > 0.05 to *p* < 0.10 difference.

Table S6 presents body composition results, while Figure 5 shows mean changes from the baseline in body composition data. Overall, multivariate analysis revealed a significant time (*p* < 0.001, η_p_^2^ = 0.234), while no interaction effects were observed (*p* = 0.647, η_p_^2^ = 0.035). Univariate analysis indicated significant time effects in weight loss (*p* < 0.001, η_p_^2^ = 0.123), fat loss (*p* < 0.001, η_p_^2^ = 0.327), and percent body fat (*p* < 0.001, η_p_^2^ = 0.348), while lean tissue mass (LTM) increased over time (*p* < 0.001, η_p_^2^ = 0.102), with no significant time effect seen in bone mineral content (BMC) or percent body water. Within-subject analysis revealed that LTM increased from the baseline in the Cr-800 group, while fat loss was achieved in all groups. However, no significant group × time interaction effects were observed. Figure 4 shows the mean change results. LTM significantly increased above the baseline in the PLA, PE-1000, and Cr-800 groups, with gains in the Cr-800 group tending to be greater than those in the PE-1000 and Cr-400 groups after 6 weeks of training, and gains in the Cr-800 group were significantly greater than those in the Cr-400 group and tended to be greater than those in the PE-500 group after 12 weeks. All groups lost fat mass, with changes in the Cr-800 group tending to be greater than those in the PLA group after 6 weeks. Fat loss was greater in the PE-500 and Cr-800 groups, with the latter tending to be greater than that in the PLA group after 6 weeks. Similarly, all groups lost percent body fat, but those in the Cr-800 group lost significantly more weight after 6 weeks than the PLA group and tended to lose more body fat than those in the PE-1000 and Cr-400 groups. At 12 weeks, those in the PE-1000 and Cr-800 groups lost a similar amount of body fat, which tended to be greater than the Cr-400 group. However, significant differences from PLA were only observed with Cr-800 supplementation after 6 weeks of supplementation. These findings indicate that Cr-800 supplementation may promote greater loss of body fat during the first six weeks of exercise training and diet intervention than PLA and Cr-400 supplementation, and it may take longer for PE-1000 to significantly affect body composition.

### 4.8. Blood Analysis

#### 4.8.1. Whole-Blood Analysis

Whole-blood red and white blood cell data are presented in Table S9. Multivariate analysis revealed a significant time (*p* < 0.001, η_p_^2^ = 0.137), while group × time effects tended to interact (*p* = 0.056, η_p_^2^ = 0.080). Univariate analysis indicated significant time effects in white blood cells (*p* = 0.009, η_p_^2^ = 0.045) and mean corpuscular volume (*p* = 0.015, η_p_^2^ = 0.041), with a significant interaction effect observed among groups in white blood cells (*p* = 0.016, η_p_^2^ = 0.086), while mean corpuscular volume (*p* = 0.081, η_p_^2^ = 0.066) and eosinophils (*p* = 0.054, η_p_^2^ = 0.074) tended to interact. Pairwise comparisons revealed some small differences among groups or during training. However, all values remained within normal clinical ranges, indicating that the ingestion of these supplements at the dosages studied was well tolerated.

#### 4.8.2. Markers of Catabolism

Table S10 presents serum markers of catabolism. Multivariate analysis revealed a significant time effect (*p* < 0.001, η_p_^2^ = 0.182), with no group × time effects (*p* = 0.943, η_p_^2^ = 0.066). Univariate analysis identified significant time effects in creatinine (*p* = 0.034, η_p_^2^ = 0.033), estimated glomerular filtration rate (*p* = 0.003, η_p_^2^ = 0.057), total protein (*p* = 0.023, η_p_^2^ = 0.036), globulin (*p* = 0.018, η_p_^2^ = 0.039), albumin/globulin ratio (*p* = 0.043, η_p_^2^ = 0.031), total bilirubin (*p* = 0.001, η_p_^2^ = 0.073), and alanine aminotransaminase (ALT, *p* = 0.011, η_p_^2^ = 0.044). However, an interaction effect was only observed in ALT value, with PE-500 values at 12 weeks tending to be lower than PE-1000 and Cr-400 values. All values remained within the expected values for trained individuals. hsCRP increased in the PE-500 above the baseline and was significantly higher than that in the Cr-400 and Cr-800 groups after 12 weeks, which would suggest greater inflammation with 12 weeks of PS-500 supplementation. However, no significant group × time effects were observed (*p* = 0.238, η_p_^2^ = 0.050).

#### 4.8.3. Blood Lipid Results

Table S11 presents serum blood lipid results. Multivariate analysis revealed a significant time effect (*p* < 0.022, η_p_^2^ = 0.056), while group × time effects tended to interact (*p* = 0.600, η_p_^2^ = 0.035). Univariate analysis indicated significant time effects in total cholesterol (*p* = 0.034, η_p_^2^ = 0.032), very-low-density lipoproteins (VLDL, *p* = 0.001, η_p_^2^ = 0.061), triglycerides (*p* = 0.012, η_p_^2^ = 0.042), and the cholesterol/HDL ratio (*p* = 0.009, η_p_^2^ = 0.044), with no significant group × time interactions. Pairwise comparisons revealed some differences in LDL cholesterol and the cholesterol/HDL ratio after 6 weeks of intervention in the PE-500 group compared to the Cr-400 group. Still, differences were small and were not retained after 12 weeks. Figure 5 presents mean change data. VLDL levels at 6 weeks in the PE-500, PE-1000, Cr-400, and Cr-800 groups were significantly lower than PLA values. Similarly, triglyceride levels at 6 weeks were significantly lower in the Cr-400 group compared to PLA values and tended to be lower in the PE-500 group. These findings indicate that PE and Cr supplementation (at both doses) may augment exercise- and diet-induced changes in VLDL and triglyceride levels after 6 weeks of supplementation, but these differences are not significantly different than PLA after 12 weeks.

#### 4.8.4. Glucose and Insulin Results

**Figure 6:**
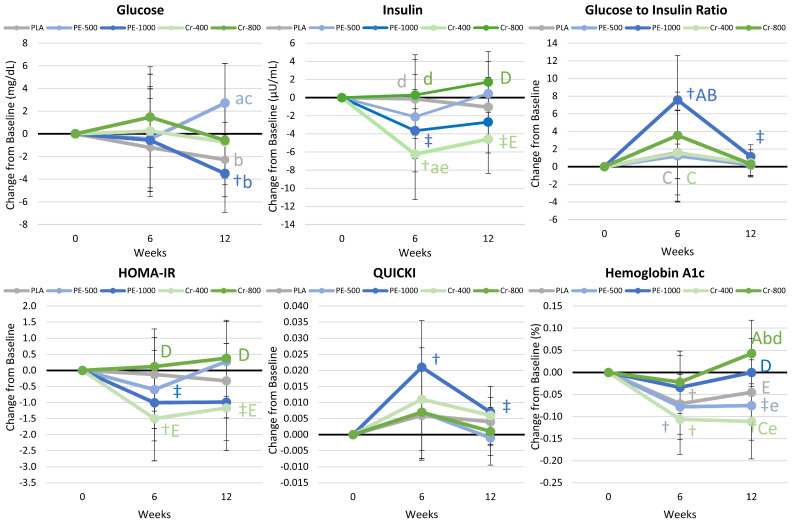
Changes in glucose homeostasis data. Data are means and 95% confidence intervals. HOMA-IR represents homeostatic model insulin resistance. QUICKI represents the quantitative insulin sensitivity check index. † is a *p* < 0.05 difference from the baseline (‡ = *p* > 0.05 to *p* < 0.10 difference). PLA = A, PE-500 = B, PE-1000 = C, Cr-400 = D, and Cr-800 = E. Lowercase letters = *p* < 0.05 difference. Uppercase letters = *p* > 0.05 to *p* < 0.10) difference.

Table S12 presents results on glucose and insulin sensitivity. Multivariate analysis revealed a significant time effect (*p* < 0.004, η_p_^2^ = 0.070), with no significant group × time effects (*p* = 0.650, η_p_^2^ = 0.035). Univariate analysis indicated significant differences or tendencies toward significance in insulin (*p* = 0.054, η_p_^2^ = 0.028), the GIR (*p* = 0.008, η_p_^2^ = 0.063), HOMA-IR (*p* = 0.067, η_p_^2^ = 0.026), QUICKI (*p* = 0.002, η_p_^2^ = 0.069), and HbA1c (*p* = 0.001, η_p_^2^ = 0.065), with no significant group × time interactions. Pairwise comparisons revealed an increase from the baseline in GIR in the PE-1000 group, with no other differences observed among groups. Figure 6 presents mean change data. Glucose levels significantly decreased after 12 weeks in the PE-1000 group, while insulin levels decreased significantly in the Cr-400 group. The GIR and QUICKI increased after 6 weeks in the PE-1000 group, while HOMA-IR decreased from the baseline with Cr-400 and PE-1000 supplementation. HbA1c decreased below the baseline in the PLA, PE-500, and Cr-400 groups after 6 and/or 12 weeks, and tended or was significantly lower than for the PE-1000 and/or Cr-800 groups. Changes in insulin levels were significantly greater than PLA in the Cr-400 group after 6 weeks. These findings indicate that PE-1000 and lower-dose Cr-400 supplementation may have greater effects on glucose and insulin homeostasis.

#### 4.8.5. Cytokines and Markers of Inflammation

**Figure 7:**
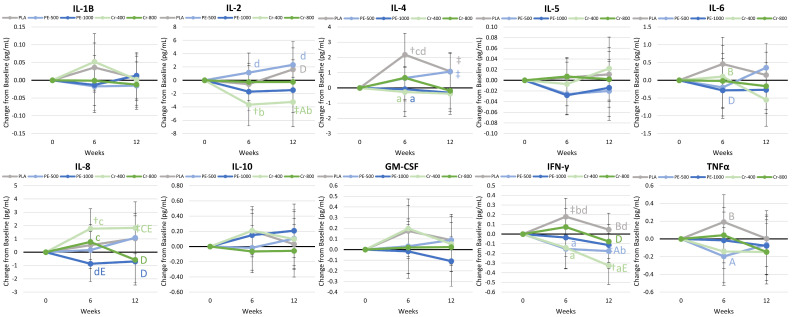
Changes in cytokines and inflammatory markers. Data are means and 95% confidence intervals. IL is interleukin, GM-CSF is granulocyte-macrophage colony-stimulating factor, IFN-γ = interferon gamma, TNFα is tumor necrosis factor alpha, † is a *p* < 0.05 difference from the baseline (‡ = *p* > 0.05 to *p* < 0.10 difference), PLA = A, PE-500 = B, PE-1000 = C, Cr-400 = D, and Cr-800 = E. Lowercase letters = *p* < 0.05 difference. Uppercase letters = *p* > 0.05 to *p* < 0.10 difference.

Table S13 presents cytokine and inflammatory marker analysis. Multivariate analysis revealed a significant time effect (*p* = 0.023, η_p_^2^ = 0.082), with no group × time effects (*p* = 0.504, η_p_^2^ = 0.048). Univariate analysis also revealed a time effect for IFN-γ (*p* = 0.004, η_p_^2^ = 0.052), with no group × interaction effects observed in this or other variables. Figure 7 presents mean change data. There was some evidence that IL-2, IL-4, IL-6, IL-10, IFN-γ, and TNFα levels were lower in supplemented groups after 6 and/or 12 weeks of training, dieting, and supplementation. IL-4 and IFN-γ levels in the PE-1000 and Cr-400 groups were significantly lower than PLA values. The results provide some support to contentions that PE supplementation with and without trivalent chromium during exercise training and diet intervention may have some effects on cytokines and markers of inflammation.

### 4.9. Platelet Aggregation

**Figure 8:**
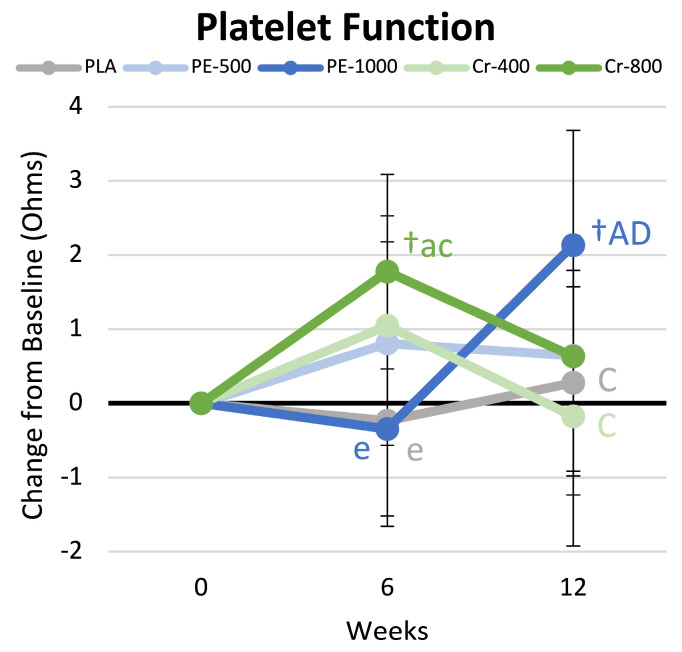
Changes in platelet aggregation values. Data are means and 95% confidence intervals. † is a *p* < 0.05 difference from the baseline. PLA = A, PE-1000 = C, Cr-400 = D, and Cr-800 = E. Lowercase letters = *p* < 0.05 difference. Uppercase letters = *p* > 0.05 to *p* < 0.10) difference.

Table S14 presents platelet aggregation data. Univariate analysis revealed a significant group × time effect (*p* < 0.022, η_p_^2^ = 0.083). Post hoc analysis revealed that platelet aggregation increased from the baseline in the Cr-800 group, which was significantly higher than PLA values and tended to be higher than PE-500 and Cr-400 values at week 6. After 12 weeks, platelet aggregation was increased in the PE-1000 group and was significantly greater than values in the PLA, Cr-400, and Cr-800 groups. Figure 8 shows the mean changes observed in platelet aggregation results. Platelet aggregation increased above the baseline in the Cr-800 group at 6 weeks and was significantly greater than values seen in the PLA and PE-1000 groups. After 12 weeks, platelet aggregation levels significantly increased above the baseline in the PE-1000 group and tended to be higher than those in the PLA and Cr-400 groups. An increase in platelet aggregation is associated with an improvement in platelet function. Consequently, the increase in platelet aggregation values observed after 6 weeks of supplementation with Cr-800 and values approaching significance after 12 weeks of supplementation in the PE-1000 group suggest that higher-dose trivalent chromium and PE supplementation may benefit platelet function.

### 4.10. Pulse Wave and Flow-Mediated Dilation Results

**Figure 9:**
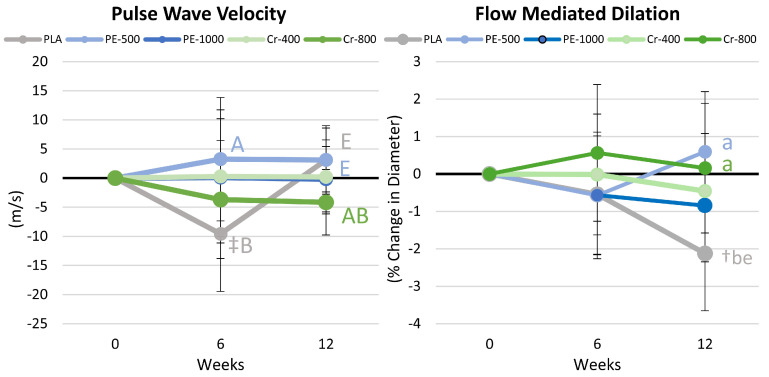
Changes in pulse wave velocity and flow-mediated dilation results. Data are means and 95% confidence intervals. † is a *p* < 0.05 difference from the baseline (‡ = *p* > 0.05 to *p* < 0.10 difference). PLA = A, PE-500 = B, and Cr-800 = E. Lowercase letters = *p* < 0.05 difference. Uppercase letters = *p* > 0.05 to *p* < 0.10 difference.

Table S15 presents the results of pulse wave velocity and FMD assessment. Multivariate analysis revealed no significant time effect (*p* = 0.464, η_p_^2^ = 0.009) or group × time effects (*p* = 0.334, η_p_^2^ = 0.041). No time or group × interaction effects were observed in univariate analysis. Figure 9 presents mean change data. After 6 weeks, PWV tended to be higher in the PE-500 group than the PLA group, while after 12 weeks, PWV tended to be higher in the PLA and PE-500 groups compared to the Cr-800 group. FMD was significantly higher after 12 weeks in the PE-500 and Cr-800 groups compared to the PLA group. Since an increase in FMD suggests greater function, the significantly greater FMD diameter observed with PE-500 and Cr-800 than with PLA supplementation suggests some benefit.

### 4.11. Profile of Mood States Results

Table S16 shows the Profile of Mood States data. GLM multivariate analysis revealed a time effects (*p* < 0.001, η_p_^2^ = 0.171) but no group × time effects (*p* = 0.935, η_p_^2^ = 0.027). Univariate analysis revealed time effects in tension (*p* = 0.037, η_p_^2^ = 0.031), depression (*p* = 0.023, η_p_^2^ = 0.037), fatigue (*p* < 0.037, η_p_^2^ = 0.128), confusion (*p* = 0.007, η_p_^2^ = 0.050), vigor (*p* < 0.001, η_p_^2^ = 0.285), and total mood disturbance scores (*p* < 0.001, η_p_^2^ = 0.138). However, no significant interaction effects were observed among groups in terms of POMS variables. Figure S3 presents mean change data. Ratings of fatigue significantly decreased from the baseline in the PLA, PE-500, and Cr-400 groups, with 6-week values tending to differ between the PE-500 and PE-1000 groups. However, no other differences were observed among the groups or in comparison to the PLA group. All groups experienced a reduction in total mood disturbance, with no differences observed among groups. These findings indicate that PE and trivalent chromium supplementation with PE do not affect mood states during an exercise and diet intervention.

### 4.12. Quality of Life, Diet Satisfaction, and Perceptions of Side Effects

Table S17 presents a Chi-squared analysis of QOL measures. Perceptions that health status limits the ability to perform vigorous activities (*p* = 0.098) and walking several hundred yards (*p* = 0.059) tended to differ among groups at the baseline, but not after 6 and 12 weeks. Perceptions about the amount of time feeling lots of energy (*p* = 0.064) and the amount of time feeling worn out (*p* = 0.091) tended to differ among groups at 6 weeks. However, no other significant differences or tendencies toward significance were observed in QOL ratings at week 6 or 12. These findings indicate that PE and trivalent chromium with PE supplementation do not promote greater changes in measures of quality of life compared to exercise and diet alone. Table S18 shows diet satisfaction questions. A significant time effect (*p* < 0.001, η_p_^2^ = 0.290) but no group × time effects (*p* = 0.295, η_p_^2^ = 0.041) were observed in GLM multivariate analysis. Significant time effects were observed in ratings of appetite (*p* = 0.051, η_p_^2^ = 0.032), energy (*p* < 0.001, η_p_^2^ = 0.366), and diet quality (*p* < 0.001, η_p_^2^ = 0.240), and a significant interaction effect was observed in hunger ratings (*p* = 0.047, η_p_^2^ = 0.072). The pairwise comparison revealed that most differences among groups were seen in the baseline assessment, while adherence to the diet promoted a more consistent rating among groups in diet satisfaction variables. These findings indicate that PE and trivalent chromium with PE supplementation did not affect appetite or perceptions about diet satisfaction. Table S19 presents the frequency and severity of reported side effects. No significant differences were reported in the frequency or severity of side effects. However, a tendency toward significance (*p* = 0.052) was observed at week 12 in the severity of dizziness, with three more participants reporting minimal and one participant rating slight severity of dizziness, with most of the change observed in the PE-1000 group. Whether this was related to PE ingestion, exercise training, other factors, or not is unclear. Nevertheless, these findings indicate that the supplements investigated were generally well tolerated.

## 5. Discussion

Exercise and nutritional interventions that promote fat loss and help manage blood glucose and lipids are often recommended as an initial means with which to reduce the risk of cardiometabolic disease before pharmacological intervention [1,2]. *Phyllanthus emblica,* Shilajit, and/or chromium supplementation have been reported to have antioxidant [6,7,8,9], anti-inflammatory [10,17,19,23], anti-diabetic [10,13,14,25,26,27,28,29,30,31,32,33], lipid-lowering [6,10,13,18], and endothelial-function-enhancing [8,13] properties that would theoretically augment the benefits of an exercise and diet intervention designed to improve fitness and health while promoting fat loss in individuals at risk for MetSyn. Additionally, co-ingesting trivalent chromium may have some additive benefits to PE and SJ supplementation. This study examined whether dietary supplementation of these nutrients in sedentary and overweight men and women with at least two risk factors of MetSyn, initiating an exercise and weight loss program, would result in more favorable changes in cardiovascular health, fitness, and weight loss markers. We found evidence that supplementing the diet with these nutrients significantly improved and/or approached significant improvements in some measures of endothelial function, platelet aggregation, insulin sensitivity, and blood lipid profiles while promoting more optimal changes in body composition, strength, and aerobic capacity than diet and exercise alone. While some benefits were seen at both dosages, the ingestion of higher dosages more consistently promoted greater effects. The following discusses primary and secondary outcomes, study limitations, and future research recommendations.

### 5.1. Primary Outcomes

#### 5.1.1. Endothelial Function and Platelet Aggregation

*Phyllanthus emblica* has been reported to increase nitrous oxide (NOS) levels [7,11,12], thereby improving endothelial function [8,93] and reducing platelet aggregation [13]. PE, chromium, and SJ also have antioxidant properties that can help scavenge superoxide and hydroxyl radicals and increase antioxidant enzymes [17,93,94,95]. A reduction in NOS and oxidative stress have been suggested to contribute to endothelial and platelet dysfunction. Consequently, the dietary supplementation of PE, chromium, and/or SJ could improve endothelial and platelet function by increasing NOS and/or reducing oxidative stress. In support of this contention, Usharani et al. [8] evaluated the effects of *Phyllanthus emblica* supplementation (500 and 1000 mg/d for 12 weeks) compared to the lipid medication atorvastatin and a placebo on markers of endothelial dysfunction in patients with type 2 diabetes. The researchers found that *Phyllanthus emblica* supplementation (both doses) improved endothelial function and reduced markers of inflammation and oxidative stress compared to placebo. Additionally, the higher dose was as effective as the medication atorvastatin in improving these markers. Khanna and coworkers [13] reported that dietary supplementation of *Phyllanthus emblica* (500 mg/d for 12 weeks) in overweight and class I obese adults significantly downregulated ADP- and collagen-induced platelet aggregation. Das and associates [96] reported that Shilajit supplementation (125 and 250 mg/d for 12 weeks) in middle-aged women improved skin perfusion and induced genes related to endothelial cell migration and blood vessel growth. Furthermore, Imanparast et al. [95] reported that chromium picolinate (500 µg/d) supplementation (with and without vitamin D_3_ (50,000 IU/week) for 16 weeks improved markers of endothelial dysfunction (vascular cell adhesion molecule-1 and plasminogen activator inhibitor-1).

In the present study, we evaluated endothelial function by assessing changes in pulse wave velocity and flow-mediated dilation. Higher pulse wave velocity values generally indicate greater arterial stiffness, while lower values are indicative of greater elasticity and healthier vessels [97]. After 6 weeks of supplementation, pulse wave velocity tended to decrease from baseline in the placebo group and was lower than PE-500 values, while values were not changed from baseline in the supplemental groups. After 12 weeks of exercise, diet, and nutritional supplementation, pulse wave velocity tended to be lower in the Cr-800 group compared to PLA and PE-500 values. Although not statistically significant, these findings indicate that higher doses of chromium supplementation during exercise and diet-induced weight loss programs may provide a greater benefit to arterial elasticity. Flow-mediated dilation represents a non-invasive method to measure vascular endothelial function and endothelium-derived nitric oxide (NO) bioavailability [60,61]. An increase in diameter after occlusion represents a more robust functional response. In the present study, after 12 weeks, the change in vessel diameter in the placebo group significantly decreased below the baseline. While the reduction was small (about 2.1%), it was significantly lower than the change in diameters observed in the PE-500 and Cr-800 groups. Although more research is necessary to replicate these findings and ascertain the specific mechanisms of action, these findings support contentions that *Phyllanthus emblica* and chromium supplementation may improve endothelial function.

A platelet aggregation study involves assessing the ability of platelets to aggregate in response to several agonists [98]. An increase in platelet aggregation to agonists demonstrates improved platelet function, while reductions in platelet aggregation are associated with several platelet-related dysfunction pathologies [98]. In the present study, platelet aggregation increased above the baseline in the Cr-800 group at 6 weeks and was significantly greater than values seen in the PLA and PE-1000 groups. After 12 weeks, platelet aggregation levels approached significance for the PE-1000 group compared to the PLA and Cr-400 groups. These findings provide some evidence that chromium, PE, and/or SJ supplementation may improve the ability of platelets to respond to several agonists. However, changes were small and varied over time, and more research is needed to replicate these findings and determine the specific mechanisms of action.

#### 5.1.2. Glucose Homeostasis and Insulin Sensitivity

Chromium supplementation has been reported to improve insulin sensitivity and glucose management, particularly in type 2 diabetic populations [30,34,35,99,100]. The mechanisms have been attributed to the role of chromium in facilitating glucose and amino acid transport as well as intracellular signaling pathways via GLUT4 translocation [101]; improving insulin binding, receptor number, beta cell sensitivity, and insulin receptor enzymes that improve insulin sensitivity [102]; and reducing reactive oxygen species (ROS), thereby reducing insulin resistance [103]. For example, Kim et al. [30] reported that chromium chloride supplementation (400 µg/d for 6 weeks) improved insulin sensitivity and body composition in overweight children. Farrokhian and coworkers [35] found that chromium supplementation (200 µg/d for 12 weeks) significantly decreased fasting glucose and insulin resistance in patients with type 2 diabetes and coronary heart disease. Alkalidi [34] reported that chromium supplementation (200 µg/d for 12 weeks) significantly decreased blood glucose and HbA1C. Moreover, a meta-analysis of 28 clinical trials by Huang and colleagues [100] found that chromium supplementation significantly reduced fasting glucose and HbA1C in type 2 diabetics regardless of the dose and length of supplementation. Conversely, other studies reported no significant effects of chromium supplementation (500–1000 µg/d for 8–36 weeks) on fasting glucose, HbA1c, and/or insulin sensitivity [25,26]. Although more limited, there are also reports that *Phyllanthus emblica* supplementation may enhance glucose management, due, in part, to PE’s antioxidant properties [8,10,13,14]. SJ has also been reported to possess antioxidant properties [17], which may also help reduce insulin resistance. In the present study, we found evidence that PE-1000, Cr-400, and Cr-800 lowered blood glucose, HbA1c, fasting insulin, and/or HOMA-IR while increasing the GIR and QUICKI after 6 and/or 12 weeks. Although benefits were observed in each group, the PE-500 and Cr-400 groups promoted significantly lower hemoglobin A1c levels after 12 weeks of supplementation than higher-dose trivalent chromium supplementation. However, the only statistically significant difference observed from the PLA group was a lower insulin level at 6 weeks of supplementation with Cr-400 and a higher GIR observed in the PE-1000 group that approached significance compared to the PLA group at 6 weeks. While the present findings provide some support to *Phyllanthus emblica* and chromium supplementation during an exercise- and diet-induced weight loss program, the results were inconsistent over time and not dose-related.

#### 5.1.3. Blood Lipids

Several studies have reported that *Phyllanthus emblica* and Shilajit supplementation can lower blood lipid levels [10,13,18,104]. Proposed mechanisms of action include reducing the absorption of cholesterol [105], inhibiting β-hydroxy β-methylglutaryl-CoA (HMG-CoA) reductase activity, thereby reducing the synthesis of cholesterol [106], and increasing lecithin cholesterol acyltransferase activity (LCAT), which plays a role in HDL metabolism [33,106,107]. For example, Sharma et al. [104] reported that Shilajit (2 g/d for 45 days) decreased triglycerides as well as total cholesterol and increased HDL cholesterol supplementation in healthy individuals. Khanna and associates [13] reported that *Phyllanthus emblica* supplementation (500 mg/d for 12 weeks) decreased LDL cholesterol and the ratio of total cholesterol to HDL cholesterol. Moreover, a meta-analysis conducted by Setayesh and colleagues [10] revealed that *Emblica Officinalis* (Amla) supplementation significantly reduced LDL cholesterol, total cholesterol, and triglycerides, while increasing HDL cholesterol. In the present study, total cholesterol significantly decreased below the baseline at 6 weeks with PE-500 supplementation, while *Phyllanthus emblica* and chromium supplementation (both doses) promoted a reduction in VLDL at week 6, while triglycerides were significantly lower in the Cr-400 group compared to the PLA group. The total cholesterol/HDL ratio tended to decrease in the PE-500, Cr-400, and Cr-800 groups, but were not significantly different than PLA or PE-1000 values. These findings support prior findings that *Phyllanthus emblica* supplementation with chromium may improve blood lipids even when following an exercise and diet program designed to promote weight loss. However, most benefits were not significantly different than PLA after 12 weeks of supplementation, and additional research must evaluate the independent and synergistic mechanisms of action.

#### 5.1.4. Inflammatory Markers

*Phyllanthus emblica*, Shilajit, and chromium have been reported to possess anti-inflammatory properties [10,17,19,23,93,108,109]. PE is believed to possess anti-inflammatory properties by inhibiting nitric oxide, COX-1, COX-2, and 5-LOX, which are involved in pro-inflammatory synthesis [5,110]. Shilajit [111] and chromium [109] have also been reported to have anti-inflammatory properties, although the specific mechanisms of action are not clear. For example, Pingali and colleagues [17] have reported that Shilajit supplementation (250 and 500 mg/d for 48 weeks) in post-menopausal women attenuated hsCRP concentrations. Usharani et al. [93] reported that *Phyllanthus emblica* (2 × 500 mg/d for 12 weeks) significantly decreased hsCRP values. Zhao and coworkers [108] reported that chromium (400 µg/d) and magnesium (200 µg/d) supplementation for 12 weeks significantly improved hsCRP. Finally, Zhang et al. [109] conducted a meta-analysis and found that chromium supplementation (200–500 µg/d for 8–28 weeks) significantly reduced hsCRP, tumor necrosis factor alpha (TNFα), and interleukin-6 (IL-6) concentrations. In the present study, hsCRP increased in the PE-500 group and was significantly higher than the Cr-400 and Cr-800 groups after 12 weeks of training and dieting. However, we did not observe any significant differences among groups in terms of IL-6 or TNFα. These findings contradict the idea that *Phyllanthus emblica* supplementation with Shilajit may reduce inflammation. However, we also found that IL-4 (anti-inflammatory cytokine [112]) and IFN-γ (pro-inflammatory cytokine [113]) concentrations in the PE-1000 and Cr-400 groups were significantly lower than placebo values. Consequently, the effects of *Phyllanthus emblica*, Shilajit, and chromium supplementation on inflammatory markers in the present study were mixed. One possible reason is that this study evaluated the effects of *Phyllanthus emblica*, Shilajit, and chromium supplementation while participating in an exercise program that included resistance exercise and following a hypoenergetic diet designed to promote weight loss. Exercise increases pro-inflammatory and immunomodulating cytokines, particularly when following a hypoenergetic diet. Thus, the exercise and diet intervention may have masked some effects. Nevertheless, the results of this study do not support the idea that *Phyllanthus emblica*, Shilajit, and chromium supplementation reduce markers of chronic inflammation.

### 5.2. Secondary Outcomes

#### 5.2.1. Body Composition

Insulin resistance and inflammation have been associated with obesity and loss of muscle mass as one ages [41,42,67]. Chromium supplementation has been reported to promote fat loss in overweight and obese populations when taking < 400 μ/d for <12 weeks [42], but not in patients with type 2 diabetes [41]. The effects of chromium supplementation on gains in muscle mass and fat loss in healthy individuals during exercise training have been mixed [114,115,116,117,118,119]. Khanna et al. [13] reported that *Phyllanthus emblica* supplementation (500 mg/d for 12 weeks) did not affect total body weight in overweight and obese individuals. However, it has not been studied in combination with an exercise and diet intervention. Das and coworkers [20] reported that Shilajit supplementation (250 mg/d for 12 weeks) had no effects on changes in body weight, even when adding walking exercise (20 min/d at 70–75% of maximum heart rate) into the protocol for the last four weeks of supplementation. However, the exercise engagement was minimal and not evaluated in conjunction with a diet designed to promote weight loss. We hypothesized that supplementing the diet with *Phyllanthus emblica*, Shilajit, and chromium during a hypoenergetic diet and exercise program that included resistance exercise may promote additive benefits compared to exercise and diet alone.

In the present study, we found that resting energy expenditure was maintained in all groups while fat oxidation increased and carbohydrate oxidation decreased, which is consistent with the impact of exercise training on substrate utilization. Participants experienced an increase in lean tissue mass (0.88 kg) and a reduction in fat mass (−2.14 kg) and body fat (−1.74%), consistent with results from our exercise- and diet-induced weight loss intervention research that included resistance exercise [3,45,46,51,69,75,86]. When evaluating the effects of supplementation interventions, Cr-400 supplementation promoted a significant increase in resting energy expenditure after 6 weeks, but this did not persist after 12 weeks. Gains in lean tissue mass were greater in the PE-1000 and Cr-800 groups, but these changes were not greater than that in the PLA group. Fat loss decreased in all groups, with changes in the Cr-800 group tending to be greater than PLA values at week 6. Percent body fat loss was significantly greater in the Cr-800 group compared to the PLA group at week 6, and tended to be greater than the PE-1000 and Cr-400 groups. After 12 weeks, body fat percentage was reduced to the greatest degree in the PE-1000 and Cr-800 groups and tended to be greater than in the Cr-400 group. However, these differences were not significantly greater than the PLA group. These findings provide some evidence that *Phyllanthus emblica*, Shilajit, and chromium supplementation during an exercise- and diet-induced weight loss program that includes resistance exercise may positively affect body composition. However, in most instances, this was not significantly greater than the placebo group. More research is needed to replicate these findings and determine the specific mechanisms of action.

#### 5.2.2. Training Adaptations

If *Phyllanthus emblica*, Shilajit, and/or chromium supplementation affect insulin sensitivity, carbohydrate and fat oxidation, oxidative stress, and/or inflammation, individuals involved in exercise training may be able to tolerate training to a greater degree, observe more favorable changes in body composition, and experience greater training adaptations. While a few studies have evaluated the effects of chromium supplementation during resistance training on body composition and strength [114,115,116,117,118,119], little is known about whether *Phyllanthus emblica* and/or Shilajit supplementation affects training adaptations. In the present study, participants experienced a 95% increase in total lifting volume while walking an average of 9224 ± 2232 steps/day. Although participants performed a similar amount of upper, lower, and total lifting volume during the 12-week training period, gains in 1RM bench press and leg press strength were significantly greater in the PE-1000 group. This is an interesting result and deserves additional study to assess the effects of PE-1000 supplementation on training adaptations in resistance-trained athletes. We also found that training promoted a significant increase in peak oxygen uptake (9.6%) and time to fatigue (18.7%) among groups, with the greatest changes in peak oxygen uptake (13.6%) and time to fatigue (35.0%) observed in the Cr-400 group. However, these differences were not significantly different than the placebo group (peak oxygen uptake 6.7%, time to fatigue 19.7%). More research is needed to determine if *Phyllanthus emblica*, Shilajit, and/or chromium supplementation affects training adaptations and mechanisms of action.

#### 5.2.3. Psychometric Assessment

People who experience weight loss, improvements in strength and aerobic capacity, and/or less inflammation during training often perceive less fatigue, better functional capacity, and/or quality of life. While we are not aware of studies that have evaluated whether *Phyllanthus emblica*, Shilajit, and/or chromium supplementation affects these outcomes, we hypothesized that there may be some psychological benefit if these supplements were effective. We found that fatigue ratings from the Profile of Mood States inventory significantly decreased from the baseline in the PLA, PE-500, and Cr-400 groups, with 6-week values tending to differ between the PE-500 and PE-1000 groups. However, no other differences were observed among the groups. Additionally, all groups experienced reduced total mood disturbance, with no differences observed among them. Analysis of the SF-36 quality of life inventory revealed that perceptions about the amount of time feeling lots of energy (*p* = 0.064) and the amount of time feeling worn out (*p* = 0.091) approached significance among groups at 6 weeks. However, no other significant differences or tendencies toward significance were observed in QOL ratings at week 6 or 12. Some differences were also observed in perceptions about diet quality at the baseline, which were minimized during the diet intervention, with no apparent differences observed among groups. Collectively, these findings indicate that 12 weeks of *Phyllanthus emblica*, Shilajit, and/or chromium supplementation do not appear to affect mood or perceptions about quality of life. However, more research is needed to understand how these nutrients affect perceptions of exercise tolerance and psychological aspects from exercise and weight loss interventions.

#### 5.2.4. Side Effect Assessment

No significant differences were reported in the frequency or severity of side effects. However, a tendency toward significance (*p* = 0.052) was observed at week 12 in the severity of dizziness, with three more participants reporting minimal and one additional participant rating slight severity of dizziness, with most of the change observed in the PE-1000 group. Analysis of clinical blood profiles indicated that values remained within normal limits. Overall, safety analysis revealed the supplements were well tolerated. The present findings support prior reports that trivalent (+3) chromium supplementation in doses up to 1000 μg/day for 5 years does not promote toxicity [120,121] as was speculated based on animal studies [122] and/or concerns about the toxicity of the hexavalent form (+6) of chromium.

### 5.3. Limitations and Challenges

Although this study attempted to control as many confounding variables as possible, several factors must be considered. First, data collection was initiated before the COVID-19 epidemic which, while it had no impact on the results of the study, impacted the ability to finish several participants in progress and recruit additional participants. Modifications to control infections required to enter the facility (exposure and symptom questionnaire, temperature readings, cleaning hands, wearing personal protection equipment (PPE)); precautions implemented to safely test and participate in an exercise program (e.g., COVID testing, requirements to wear masks and face shields while exercising behind plexiglass barriers and 10–20 feet away from others); apprehension from physicians to approve participation in the study; and participants’ concerns about being exposed to others during testing and training sessions impacted interest in participating in the study, compliance, and the time it took to complete the study. Our study cohort was limited to men and women living around a university clinical research facility who were willing to participate in a supervised exercise program and diet intervention program, were overweight, and had at least two risk factors for MetSyn, who were not being pharmacologically treated. While this allowed for an assessment of pre-MetSyn participants, finding individuals with moderately high triglycerides, blood pressure, and/or fasting glucose who were not already diagnosed or being medically treated for MetSyn risk factors was challenging. Finally, this study was conducted during challenging financial times that increased transportation costs, which impacted compliance and dropout rates. Consequently, the results of this study should be viewed with these challenges in mind.

### 5.4. Future Directions

Additional research should evaluate whether *Phyllanthus emblica*, Shilajit, and/or chromium supplementation may improve training and body composition adaptations in active aging individuals initiating an exercise program to promote health with and without risk factors for MetSyn. The changes in body composition, peak oxygen uptake, 1RM strength, and insulin sensitivity observed in some of the supplemented groups should be evaluated in younger individuals and athletes involved in training. More research should be conducted on whether *Phyllanthus emblica*, Shilajit, and/or trivalent chromium supplementation can promote improvements in blood lipids without exercise training. *Phyllanthus emblica*, Shilajit, and/or chromium supplementation should also be studied in people treated for hyperlipidemia, glucose intolerance, arterial stiffness, and platelet dysfunction to see if it may reduce the need for medications to manage these conditions. Finally, additional work should determine if adding *Phyllanthus emblica*, Shilajit, and/or chromium to other nutritional approaches to promote muscle accretion, prevent sarcopenia, improve insulin sensitivity, manage blood glucose, and/or promote vascular health would offer additional benefits. This includes further elucidating the independent and synergistic mechanisms of action.

## 6. Conclusions

Within the limitations of this study, the results suggest that *Phyllanthus emblica*, Shilajit, and/or chromium supplementation may enhance some exercise- and diet-induced changes in endothelial function, platelet aggregation, insulin sensitivity, blood lipids, body composition, and training adaptations. However, these findings should be viewed as exploratory, as some potential benefits observed after 6 weeks were not sustained after 12 weeks and some differences only approached significance with moderate effect sizes. Additional research is needed to evaluate how *Phyllanthus emblica*, Shilajit, and/or chromium supplementation may affect training adaptations in healthy and active aging populations with and without MetSyn risk factors, and among individuals being medically managed for chronic disease and mechanisms of action before conclusions can be drawn.

## Data Availability

Data and statistical analyses are available upon request for non-commercial scientific inquiry and/or educational purposes if the request and use do not violate IRB restrictions and/or research agreement terms.

## Supplementary Materials

The following supporting information can be downloaded at: https://www.mdpi.com/article/10.3390/nu17122042/s1, Table S1: Participant demographics, Table S2: Absolute and relative energy and macronutrient intake, Table S3: Resistance training lifting volume Table S4: Average step counts reported on non-training days, Table S5: Resting energy expenditure data, Table S6: Body composition data, Table S7: Cardiovascular health and fitness results, Table S8: Muscular strength and endurance results, Table S9: Whole-blood red and white blood cell counts, Table S10: Serum markers of catabolism, Table S11: Serum blood lipid results, Table S12: Glucose and insulin results, Table S13: Cytokine and inflammatory marker analysis; Table S14: Platelet aggregation function results, Table S15: Pulse wave velocity and flow-mediated dilation data, Table S16: Profile of Mood States results, Table S17: Quality of life ratings, Table S18: Diet satisfaction analysis, Table S19: Frequency and severity of side effects, Figure S1: Resting energy expenditure change results, Figure S2: Changes in resting hemodynamics and aerobic capacity, Figure S3: Changes in muscular strength and endurance, Figure S4: Changes in Profile of Mood States.

## Abbreviations

The following abbreviations are used in this manuscript:

1RM	One-repetition maximum
ANOVA	Analysis of variance
BMC	Bone mineral content
BMI	Body mass index
CI	Confidence interval
CONSORT	Consolidated Standards of Reporting Trials
Cr	Chromium
Cr-400	Chromium (400 μ/d) + 6 mg/d PE + 6 mg/d SJ
Cr-800	Chromium (800 μ/d) + 12 mg/d PE + 12 mg/d SJ
DEXA	Dual-energy X-ray absorptiometer
DOAJ	Directory of open access journals
GIR	Glucose/insulin ratio
GLM	General linear model
GMC-SF	Granulocyte-macrophage colony-stimulating factor
GPXT	Cardiopulmonary exercise test
HDL	High-density lipoprotein
HOMA-IR	Homeostatic model assessment for insulin resistance
HRR	Heart rate reserve
IFN-γ	Interferon-γ
IL	Interleukin
LDL	Low-density lipoproteins
LSD	Least significant difference
LTM	Lean tissue mass
MDPI	Multidisciplinary Digital Publishing Institute
PE	*Phyllanthus emblica*
PE-1000	*Phyllanthus emblica* (1000 mg/d)
PE-500	*Phyllanthus emblica* (500 mg/d)
PLA	Placebo
POMS)	Profile of Mood States
QOL	Quality of life
QUICKI	Quantitative insulin sensitivity check index
SBP	Systolic blood pressure
DBP	Diastolic blood pressure
SD	Standard deviation
SJ	Shilajit
TNF-α	Tumor necrosis factor-α
VLDL	Very-low-density lipoproteins
